# Improved Bio-Synthesis of 2,5-bis(hydroxymethyl)furan by *Burkholderia contaminans* NJPI-15 With Co-substrate

**DOI:** 10.3389/fchem.2021.635191

**Published:** 2021-02-03

**Authors:** Siyuan Chang, Xuejun He, Bingfeng Li, Xin Pan

**Affiliations:** ^1^ School of Biology and Environment, Nanjing Polytechnic Institute, Nanjing, China; ^2^ Department of Cardiology, The Affiliated Hospital of Yangzhou University, Yangzhou University, Yangzhou, China

**Keywords:** 5-Bis(hydroxymethyl)furan, bio-synthesis, Burkholderia contaminans NJPI-15, fed-batch strategy, co-substrate

## Abstract

Upgrading of biomass derived 5-hydroxymethylfurfural (HMF) has attracted considerable interest recently. A new highly HMF-tolerant strain of *Burkholderia contaminans* NJPI-15 was isolated in this study, and the biocatalytic reduction of HMF into 2,5-bis(hydroxymethyl)furan (BHMF) using whole cells was reported. Co-substrate was applied to improve the BHMF yield and selectivity of this strain as well as HMF-tolerant level. The catalytic capacity of the cells can be substantially improved by Mn^2+^ ion. The strain exhibited good catalytic performance at a pH range of 6.0–9.0 and a temperature range of 25°C–35°C. In addition, 100 mM HMF could be reduced to BHMF by the *B. contaminans* NJPI-15 resting cells in presence of 70 mM glutamine and 30 mM sucrose, with a yield of 95%. In the fed-batch strategy, 656 mM BHMF was obtained within 48 h, giving a yield of 93.7%. The reported utilization of HMF to produce BHMF is a promising industrially sound biocatalytic process.

## Introduction

Increasing the availability of renewable raw materials to reduce the use of fossil fuels has recently become a major research interest (Chang et al., 2017; Zhang et al., 2019). It was well known that 5-hydroxymethylfurfural (HMF) could be synthesized from lignocellulose and was one of the “Top 10 + 4” bio-based platform chemicals (Bozell and Petersen, 2010; Zhang et al., 2017). Because of the presence of three functional groups, including an aldehyde group, a hydroxyl group and a furan ring, it shows a very strong reactivity (Hu et al., 2018a). As a result, HMF could be transformed into a variety of valuable products such as 5-hydroxymethyl-2-furancarboxylic acid (HMFCA), 2,5-diformylfuran (DFF), 2,5-bis(hydroxymethyl)furan (BHMF) and 2,5-furandicarboxylic acid (FDCA) (Cang et al., 2019; Lăcătuş et al., 2020). BHMF is the hydrogenation product of the formyl group in HMF and is a versatile building block for the synthesis of polymers, drugs, and macrocycle polyether compounds. Additionally, it is known that fatty acid diesters of BHMF have applications as biodiesel additives and nonionic surfactants (Petri et al., 2018; Xu et al., 2018; Wang et al., 2020).

Chemical approaches currently play a dominant role in the production of BHMF from HMF (Kong et al., 2018). Particularly, considerable effort has been dedicated to the development of a variety of metal catalysts for selective hydrogenation of HMF to BHMF (Lăcătuş et al., 2018). Biocatalysis is emerging as an additional pillar for green and clean valorization of HMF, due to increased chemical and regional selectivity as well as mild reaction conditions in aqueous environments (Cang et al., 2019; Wang et al., 2019). Biocatalytic reduction of HMF to BHMF with whole cells is still a great challenge because the substrate HMF is a well-known potent inhibitor to microorganisms (Shi et al., 2019; Zhang et al., 2020). Thus, the reported on biocatalytic synthesis of BHMF from HMF remain limited in the literature. Li et al. (2017) reported that a HMF-tolerant *Meyerozyma guilliermondii* SC1103 for the synthesis of BHMF; a good yield and an excellent selectivity were obtained in 12 h, when the substrate concentration was 100 mM. Furthermore, they identified alcohol dehydrogenases from the SC1103 and were heterologously expressed in *S. cerevisiae* for the synthesis of 250 mM BHMF from HMF (Xia et al., 2020). He et al. (2018) found that the recombinant *Escherichia coli* CCZU-K14 displayed a higher HMF-tolerant level as well as good catalytic activities in the HMF reduction; BHMF was afforded with a high yield of approximately 91% in 72 h when the substrate concentration was up to 200 mM. Although good results were achieved recently reported, the substrate concentration for biocatalytic reduction of HMF was usually unsatisfied, resulting in the biocatalyst low productivity. Therefore, improving the HMF-tolerance of microbes and their catalytic activity is an important approach for improving biocatalysts yield of BHMF.

Except from natural environment, stimulated potential ability of the microbe was another good way (Li et al., 2012). Co-substrate auxiliary strategy established in environmental microbiology was applied to the biosynthesis of other high-value chemicals (Polet and Feron, 2013). He et al. (2015) reported that adding L-glutamine and glycine to the reaction media improved the NADH-dependent COBE-reductase activity 1.6-fold compared to the control without supplementation. In a study by Nie et al. (2009), the reaction conversion efficiency of the deracemization of racemic 1-phenyl-1,2-ethanediol to (S)-isomer was significantly improved by the addition of extra pentoses. Co-substrate provided additional NADH or NADPH to improve the efficiency of oxidation-reduction reactions (Ma et al., 2007). In this study, we reported the use of *Burkholderia contaminans* NJPI-15, a new HMF-tolerant strain isolated from chemically polluted soil, for the efficient synthesis of BHMF from HMF. The effects of various parameters (co-substrate, reaction pH, reaction temperature, metal ion, and substrate concentration, etc.) on the BHMF catalytic performance by this strain were investigated. A fed-batch strategy was applied to enable the accumulation of BHMF.

## Materials and Methods

### Chemical Materials

HMF were purchased from the Aladdin Co., Ltd (Shanghai, China). BHMF were purchased from Sigma–Aldrich Co., Ltd (Shanghai, China). HMFCA, MMF, DFF, and FFCA were purchased from Tokyo Chemical Industry Co., Ltd (Shanghai, China).

### General Procedure for Bioreduction of *B. contaminans* NJPI-15

*Burkholderia contaminans* NJPI-15 was isolated from chemical industrial soil samples. The strain was deposited in the China Center for Type Culture Collection (CCTCC) with the access number: M2020636. The GenBank accession number for the 16S rDNA gene sequence of the strain was MW165517. The screening medium (g/L) containing: glucose, three; peptone, five; yeast extract, 2.5 g; MgSO_4_, 0.25; KH_2_PO_4_, 1.0; HMF, 4. The *B. contaminans* NJPI-15 cells were cultivated in Luria-Bertani (LB) medium, 2% seed culture was inoculated to the fresh LB medium containing a low concentration of HMF. The cells harvested *via* centrifugation at 12,000 rpm for 10 min, washed, and finally dispersed in phosphate buffer. Several co-substrates, (i.e. glucose, glycerol, sucrose, aspartic acid, nicotinic acid, glutamine, glycine, ribose, and xylose) were tested for improved *B. contaminans* NJPI-15 bioreduction activity.

Biocatalytic experiments were performed as follows: 10 mL of phosphate buffer (50 mM, pH 7) containing different concentrations of HMF and 20 mg/mL (wet weight) *B. contaminans* NJPI-15 were incubated at 35°C and 180 rpm. The samples were collected from the reaction mixture every 2 h. Yield was defined as the ratio of the amount of formed BHMF based on the initial HMF quantity to the theoretical values. The conversion was defined as the ratio of the consumed HMF amount to the initial amount of HMF.

### Synthesis of BHMF by a Fed-Batch Strategy

The reaction mixture was incubated at 35°C and 180 rpm, containing 50 mM pH 7 PBS, 100 mM HMF, co-substrate (70 mM glutamine, 30 mM sucrose), 1.0 mM Mn^2+^, and 20 mg/mL (wet weight) *B. contaminans* NJPI-15 resting cells. When the HMF was almost used up, 100 mM HMF and co-substrates were supplied. After 33 h, the cells were separated from the reaction mixture and added to a fresh mixture of 50 mM PBS, 100 mM HMF and co-substrates for the next round.

### HPLC Analysis

The reaction was analyzed by high-performance liquid chromatography (HPLC) (Dionex P680 HPLC) using a SymmetryShield RP18 column (4.6 mm × 250 mm, Kromasil, Sweden). The maximum absorption wavelengths of HMF, BHMF, and HMFCA were 283 nm, 223 nm, and 252 nm, respectively. The mobile phases were composed of 0.1% aqueous acetic acid in water (solvent A) and methanol (solvent B) in isocratic elution program (92% A: 8% B). The flow rate was set at 0.8 mL/min. The retention times of HMF, BHMF, and HMFCA were 12.1, 11.0 and 23.8 min, respectively.

## Results and Discussion

### Effects of Co-substrates on BHMF Synthesis

Because of the high toxicity of HMF, an appropriate co-substrate has proved to be crucial for enhancing the biosynthesis performance of bacteria (Shi et al., 2019; Zhang et al., 2020). The effects of co-substrates on BHMF synthesis were shown in Table 1. A slightly higher yield of BHMF was achieved after 10 h of reaction using glucose (93.6%) compared to the other co-substrates. However, the selectivity was higher for sucrose (99.1%) compared to the other co-substrates. Moreover, the prolongation of the reaction time to 16 h was almost unchanged the yield and selectivity of BHMF. To reduce the cost of separation, sucrose was selected as the best co-substrate. In addition, the HMF reduction performance of *B. contaminans* NJPI-15 markedly decreased without co-substrates. Poor yield and selectivity (55.3% and 81.8%, respectively) were obtained after 10 h in the absence of co-substrate, and prolonging the reaction time did not increase the yield or selectivity. This result illustrated that the co-substrate played a key role in the bio-catalyzed reduction of HMF, and confirmed that the product was furnished with sufficient yield after 10 h. Glucose or sucrose were the carbon source for *B. contaminans* NJPI-15 cells, they might be able to provide a reduced form of NAD(P)H for HMF bioconversion (Li et al., 2017). Moreover, the HMF reduction performance of the microbe cells decreased with increasing HMF concentration. Using 100 mM HMF led to lower yield and selectivity by *B. contaminans* NJPI-15, suggesting that sucrose could not satisfy NAD^+^ supplementation.

**TABLE 1:** Comparison of the catalytic performance of co-substrates.

	Co-substrates	Conc. Of HMF (mM)	Time (h)	BHMF yield (%)	Selectivity (%)
1	Glycerol	50	10	84.4 ± 1.2	87.1 ± 0.1
2	Glucose	50	10	93.6 ± 0.8	92.6 ± 0.2
3	Sucrose	50	10	89.3 ± 0.5	99.1 ± 0.0
4	None	50	10	55.3 ± 1.0	81.8 ± 0.1
5	Sucrose	50	16	92.8 ± 1.3	99.4 ± 0.0
6	Glucose	50	16	95.7 ± 0.8	92.7 ± 0.1
8	Sucrose	100	10	62.4 ± 0.5	88.4 ± 0.1
9	Glucose	100	10	69.4 ± 0.3	82.5 ± 0.2

Reaction conditions: 30 mM co-substrates, 20 mg/mL (wet weight) *B. contaminans* NJPI-15 cells, phosphate buffer (50 mM, pH 7.0), 35°C, 180 rpm.

**FIGURE 1:**
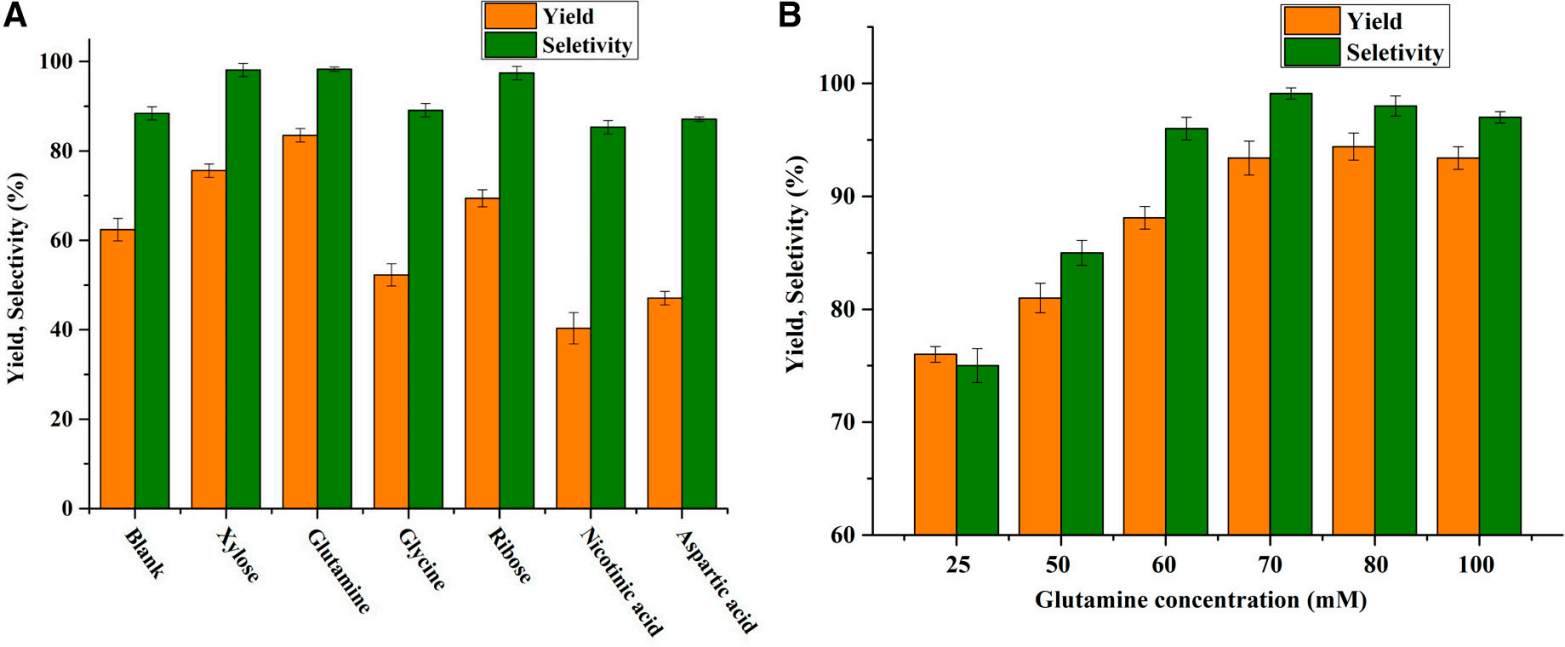
**(A)** Effect of various potential co-substrates on BHMF yield and selectivity. Reaction conditions: 100 mM HMF, 20 mg/mL (wet weight) *B. contaminans* NJPI-15 cells, 35°C, 180 rpm. **(B)** Effect of glutamine concentration on the BHMF yield and selectivity. Reaction conditions: 100 mM HMF, 20 mg/mL (wet weight) *B. contaminans* NJPI-15 cells, 35°C, 180 rpm.

To supplement sufficient NAD^+^, various types of co-substate (aspartic acid, nicotinic acid, glutamine, glycine, ribose, and xylose) were added assisted of NAD^+^ at a high concentration of HMF (100 mM). As shown in Figure 1A, glutamine was capable of enhancing the BHMF yield (83.5%) and selectivity (98.3%), achieving superior results to glycine (62.3%, 89.1%), ribose (69.4%, 98.3%), and xylose (75.6%, 98.1%). In contrast, aspartic acid and nicotinic acid decreased the yield of BHMF. Furthermore, effect of the concentration of glutamine (0–100 mM) on the HMF-reduction activity was investigated. As presented in Figure 1B, the highest yield of BHMF (95.2%) and the highest selectivity (99.1%) were obtained with a glutamine concentration of 70 mM. The yield was slightly decreased when the glutamine concentration was further increased to 100 mM. This result indicated that over 70 mM glutamine could not significantly improve BHMF yield and selectively. Therefore, the appropriate concentration of glutamine was 70 mM.

Variety type of co-substrates (glutamine, glucose, xylose, and β-cyclodextrin. etc.) have been reported to be directly/indirectly involved in regenerating the reduced cofactors (He et al., 2015; Xu et al., 2020). Glutamine plays a pleiotropic role in cell function, contributing to processes such as energy synthesis, macromolecular synthesis, mTOR activation, and reactive oxygen balance (Sayed et al., 2019). Liu et al. (2019) reported that a cofactor self-sufficient system was designed for enhanced 1,2-amino alcohols production in *E. coli* BL21 by overexpressing a glutamate dehydrogenase. Efficient biosynthesis of (S)-1,2-amino alcohols with respective high yields without addition of external cofactors and amino donors. He et al. (2015) reported that the mixture of glutamine and D-xylose instead of NAD^+^ was employed to improve the reductase activity and produce (R)-CHBE. Regarding HMF reduction, the present of glutamine significantly improved the efficiency of *B. contaminans* NJPI-15 cells transforming HMF, indicating that glutamine might directly or indirectly participate in the synthesis of cofactor NADH (He et al., 2018). Furthermore, the sucrose might increase the cofactor pool after being metabolized in the pentose phosphate pathway (Nie et al., 2009). Thus, the mixture of glutamine (70 mM) and sucrose (30 mM) was chosen as the optimum co-substrates for the following investigation.

### Effect of pH and Temperature on BHMF Synthesis

**FIGURE 2:**
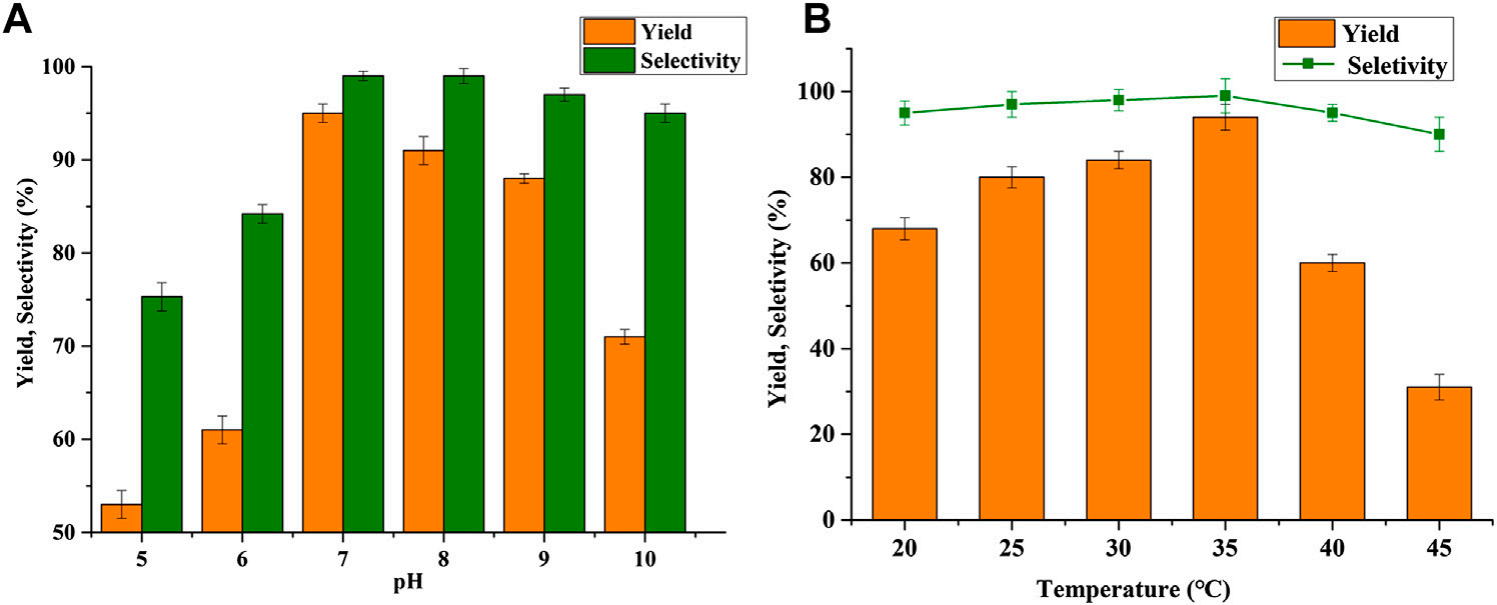
Effect of **(A)** pH and **(B)** temperature on BHMF yield and selectivity.

The effect of pH on BHMF synthesis, with the pH values varying from 5.0 to 10.0, was shown in Figure 2A. It was found that *B. contaminans* NJPI-15 exhibited good performance within the pH range of 7.0–9.0, and the highest yield of 95.1% was achieved at a pH of 7.0. The BHMF yield was around only 50% at pH values of 5.0 and 6.0, and the selectivity was also unsatisfactory at 75.3% and 84.2%, respectively. The *B. contaminans* NJPI-15 cells displayed good HMF-reduction activity within neutral or weak alkaline conditions.

The effect of reaction temperature on *B. contaminans* NJPI-15 reduction of HMF was shown in Figure 2B. The cells displayed comparable selectivity at 25°C–35°C, but only 80.4% conversion at 45°C. Furthermore, conversions of 68.1% and 37.3% were obtained at 20°C and 45°C. The highest yield and selectivity were both observed at 35°C. The temperature above 35°C, the BHMF yield and selectively decreased possibly due to the thermal deactivation of reductase in the *B. contaminans* NJPI-15. This result indicated that the relationship between reaction temperature and substrate utilization was significant.

### Effect of Substrate Concentration and Metal Ion on BHMF Synthesis

**FIGURE 3:**
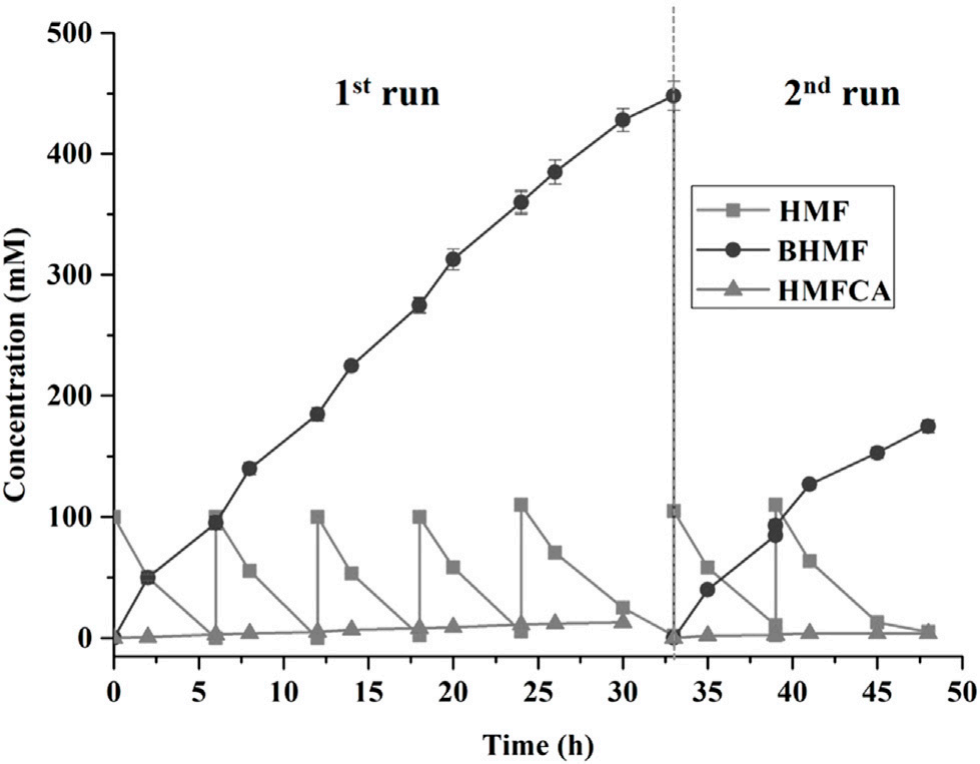
**(A)** HMF concentration on BHMF yield and selectivity. **(B)** Effect of various metal ions (1.0 mM) on BHMF synthesis. **(C)** Effect of various Mn^2+^ concentrations on BHMF synthesis. Reaction conditions: 125 mM HMF, 20 mg/mL (wet weight) *B. contaminans* NJPI-15 cells, 35°C, 180 rpm.

The success of a biocatalytic process strongly depends on the tolerance of the biocatalyst toward high substrate concentrations (Xu et al., 2018). It is widely known that the substrate HMF is a well-known inhibitor to microorganisms (Shi et al., 2019). Therefore, the biotransformation of various concentrations of HMF into BHMF using *B. contaminans* NJPI-15 (Figure 3A). It was found that the BHMF was obtained with an 85.7% yield when the HMF concentration was lower than 125 mM. Further increasing the HMF concentration resulted in significantly reduced catalytic performances of the *B. contaminans* NJPI-15 cells, possibly due to the substantially inhibitory and toxic effects of high concentrations of HMF on whole-cell biocatalysts. As shown in Figure 2A, the selectively also decreased significantly with further increasing substrate concentrations.

The dependence of metal ions is one of important features for the classification of oxidoreductase (Wang et al., 2011). Effects of various metal ions on the *B. contaminans* NJPI-15 biocatalytic activity were investigated at 35°C and pH 7.5 (Figure 3B). Compared to the control, Cu^2+^, Co^2+^, Ni^2+^and Cr^2+^ caused inhibition on the cell catalytic activity. Addition 1.0 mM Ca^2+^, Zn^2+^ and Fe^2+^ in the reaction enhanced BHMF yield. The Mn^2+^ promoted the whole cell reduction activity clearly, leading to 11% increase in BHMF yield. Thus, effects of Mn^2+^ concentrations (0–10 mM) on the BHMF synthesis were determined. As presented in Figure 3C, Mn^2+^ (0–1.0 mM) could promote HMF-reduction activity of *B. contaminans* NJPI-15. The highest biocatalytic activity was obtained at 1.0 mM Mn^2+^. At over 2.0 mM, the HMF-reduction activity decreased greatly. The role of Mn^2+^ implies that manganese ion dependent oxidoreductase inside cells may contribute to the conversion of HMF to BHMF. Nevertheless, the results need to be verified by isolation and characterization of corresponding enzyme in further work.

### Synthesis of BHMF by a Fed-Batch Strategy

To be suitable for industrial production, a high concentration of HMF must be used for BHMF production. Owing to HMF’s toxicity and inhibitory effect on microbial cells and enzymes, it was usually difficult to obtain desired products from a high concentration of HMF. Thus, induced microbial cells by HMF, furfural or furfural alcohol with low concentration during cultivation process were reported to show improved catalytic activities (Pan et al., 2020; Chen et al., 2021). This strategy proved to be effective in the *B. contaminans* NJPI-15 involved HMF conversion process. The BHMF yield was approximately 60% within 6 h in the control, whereas the 2–3 mM HMF-adapted yield were 95% obtained within the same reaction time.

**FIGURE 4:**
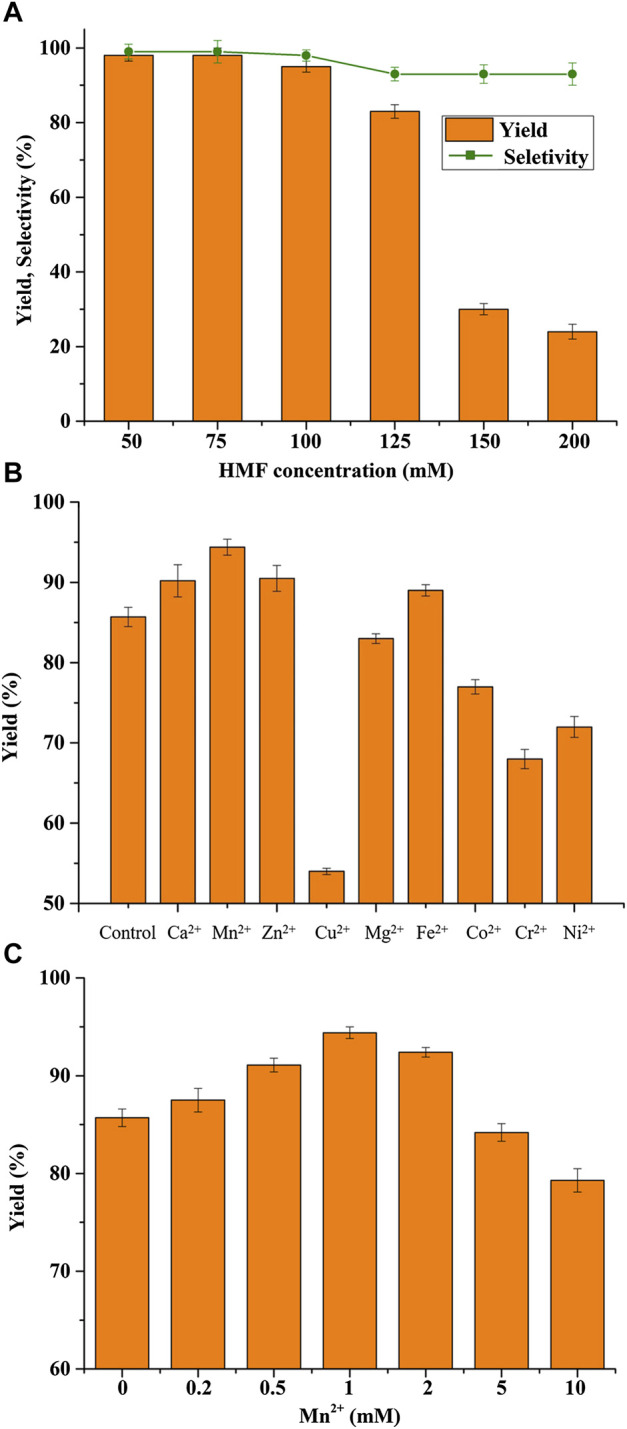
Biocatalytic synthesis of BHMF by a fed-batch strategy. Reaction conditions: 100 mM HMF, 20 mg/mL (wet weight) *B. contaminans* NJPI-15 cells, 35°C, 180 rpm. The whole-cell biocatalyst was isolated from the reaction mixture after 33 h and then added into the fresh reaction mixture for the second run. The relative activity of the intact cells was defined as 100%.

It is highly preferable that continuous accumulation of a high concentration of BHMF in the reactor for the purpose of practical applications. A fed-batch strategy for the biocatalysis of BHMF was implemented in this study. BHMF with a concentration of over 285 mM was produced after 18 h, and the complete conversion of HMF within each batch required 6 h (Figure 4). Furthermore, the reaction times were extended to 9 h after the fifth HMF feeding. The product inhibition might have partially contributed to the reduced HMF conversion with 474 mM BHMF. Thus, the cells were isolated and added to a mixture of fresh medium and HMF, in which the product inhibition was free and NADP deficiency was compensated. *B. contaminans* NJPI-15 significant deactivation of the biocatalysts after the eighth feeding. The HMF-reduction activity was only 70%. This result was possibly due to the notoriously toxic HMF, which damages the cell wall and enzymes. Finally, with the seventh reaction feed, up to 656 mM of the desired product was produced within 48 h.

Recently, a number of whole cell or enzyme biocatalysts has been discovered and applied in HMF conversion (Hu et al., 2018b). However, biocatalysts eligible for selective reduction of HMF with a high concentration for BHMF synthesis are still scarce. The biocatalytic BHMF production using whole-cell biocatalysts so far were shown in Table 2. Recombinant *E. coli* CCZU-K14 and *M. guilliermondii* SC1103 could convert 200 mM of HMF, giving excellent BHMF yield of 90.6% and 95.5%, respectively (Li et al., 2017; He et al., 2018). The low substrate concentration were not ideal for biocatalytic. In addition, BHMF yield of 84% was obtained with immobilized *M. guilliermondii* SC1103 cells when HMF concentration was 800 mM (Xu et al., 2018). Recombinant *S. cerevisiae* expressed alcohol dehydrogenase was constructed, and BHMF yield was achieved 86.3% with 400 mM of HMF after 23 h (Xia et al., 2020). *A. subglaciale* F134 was applied for BHMF synthesis with the yield of 86% in the presence of 500 mM HMF (Chen et al., 2021). Compared to previous reported, a total concentration of 656 mM BHMF was obtained from 700 mM HMF within 48 h in this study, which was the highest yield reported at high HMF concentration to date in the literature.

**TABLE 2:** BHMF biosynthesis via HMF reduction by various whole-cell biocatalysts.

Strain	HMF (mM)	Cell dosage	Shaker speed (rpm)	Yield (%)	Time (h)	BHMF (mM)	Ref
Recombinant *E. coli CCZU-K14*	200	100 mg/mL	160	90.6	72	181.2	He et al., 2018
*M. guilliermondii* SC1103	200	20 mg/mL	200	95.5	24.5	191	Li et al., 2017
Immobilization *M. guilliermondii* SC1103	800	50 mg/mL	200	84%	36	672	Xu et al., 2018
Recombinant *S. cerevisiae*	400	60 mg/mL	200	86.3	23	345	Xia et al., 2020
*A. subglaciale* F134	500	200 mg/mL	850	86	15	430	Chen et al., 2021
*B. contaminans* NJPI-15	700	20 mg/mL	180	93.7	48	656	This study

## Conclusion

In summary, an efficient biocatalytic process for the synthesis of BHMF from HMF was successfully developed using newly isolated *B. contaminans* NJPI-15. The cells were highly tolerant of HMF and proved to be an excellent biocatalyst for reduction activity on HMF with the assistance of 70 mM glutamine and 30 mM surcose, which enhanced the conversion and selectivity. BHMF up to 656 mM was achieved after 48 h with a fed-batch strategy, which was improved though cell cultivation in the presence of 2.0 mM HMF. Hence, this study demonstrates that *B. contaminans* NJPI-15 may have promising application potential in HMF bioconversion.

## Data Availability Statement

The datasets presented in this study can be found in online repositories. The names of the repository/repositories and accession number(s) can be found in the article/Supplementary Material.

## Author Contributions

XP conducted the experiments. SC and BF performed the whole-cell catalytic oxidation experiments. SC wrote the manuscript and made substantial revisions. Supervision, funding acquisition, review, and editing of the manuscript were carried out by XP and XH. All authors have approved the final version of the manuscript.

## Funding

This work was financially supported by National Natural Science Foundation of China (82000257), the Natural Science Foundation of Jiangsu (BK 20200143), Natural Science Foundation of Jiangsu Higher Education Institutions of China (20KJB180014), the Social Development Project of Yangzhou City (YZ2020087), the Startup Foundation for Advanced Talents of Yangzhou University (BS2019PX) and Nanjing polytechnic institute scientific research project (NHKY-2019-18).

## Conflict of Interest

The authors declare that the research was conducted in the absence of any commercial or financial relationships that could be construed as a potential conflict of interest.
